# Combining meta reinforcement learning with neural plasticity mechanisms for improved AI performance

**DOI:** 10.1371/journal.pone.0320777

**Published:** 2025-05-15

**Authors:** Liu Liu, Zhifei Xu

**Affiliations:** 1 College of Business Administration, Capital University of Economics and Business, Beijing, China; 2 School of Science and Engineering, Chinese University of Hong Kong - Shenzhen, Shenzhen, Guangdong, China; University of Hamburg: Universitat Hamburg, Germany

## Abstract

This research explores the potential of combining Meta Reinforcement Learning (MRL) with Spike-Timing-Dependent Plasticity (STDP) to enhance the performance and adaptability of AI agents in Atari game settings. Our methodology leverages MRL to swiftly adjust agent strategies across a range of games, while STDP fine-tunes synaptic weights based on neuronal spike timings, which in turn improves learning efficiency and decision-making under changing conditions. A series of experiments were conducted with standard Atari games to compare the hybrid MRL-STDP model against baseline models using traditional reinforcement learning techniques like Q-learning and Deep Q-Networks. Various performance metrics, including learning speed, adaptability, and cross-game generalization, were evaluated. The results show that the MRL-STDP approach significantly accelerates the agent’s ability to reach competitive performance levels, with a 40% boost in learning efficiency and a 35% increase in adaptability over conventional models.

## 1. Introduction

Artificial intelligence (AI) is progressively playing a crucial role in contemporary technology, impacting diverse sectors, including healthcare diagnostics and autonomous vehicles [1–10]. One particularly intriguing area where AI is applied is in video games—dynamic environments that allow for the testing and optimization of AI in a controlled yet complex manner. The video game industry, valued at over $159 billion in 2020 and projected to approach $300 billion by 2027, serves as a fertile ground for AI innovation [11–15]. These virtual settings offer an opportunity to simulate real-world scenarios within defined parameters, making them ideal for experimenting with and refining AI techniques. AI’s role in video games extends beyond just making games more engaging; it involves enhancing algorithms that can later be used to address real-world challenges. For example, strategies developed in gaming environments can be transferred to robotics, automated systems, and complex decision-making processes. The relevance of improving AI for gaming not only lies in enhancing user experiences but also in preparing AI systems to efficiently adapt and make decisions in unpredictable real-world situations.

Reinforcement Learning (RL) serves as a cornerstone in AI, specifically in teaching machines to learn from their environment and make strategic decisions [16–23]. The foundation of RL lies in the early studies of optimal control theory, where it was initially employed to program computers for playing checkers in the 1950s [24,25]. These advancements stemmed from the idea that an agent learns to accomplish tasks through interaction with an environment, relying on trial and error to optimize cumulative rewards. This approach has been widely applied, ranging from simple games to the more complex challenge of training software agents to autonomously navigate real-world environments.

However, traditional RL methods often struggle when faced with the complex and unpredictable nature of modern video games. Typically, these approaches begin learning from scratch, accumulating experiences to form their knowledge base, which can result in several inherent limitations:

- Slow Adaptation: Traditional reinforcement learning methods are often slow to adapt, as they require prolonged interaction with the environment to gather enough data to make accurate predictions. For example, DeepMind’s AlphaGo had to play millions of games against itself to perfect its strategy, a highly resource-intensive and time-consuming task.
- Handling Large State Spaces: Video games commonly feature vast state spaces with many interconnected variables, which traditional RL algorithms find difficult to manage. These algorithms often struggle to apply learned knowledge across different states, reducing their efficiency in environments where the state variables can shift unexpectedly.
- Generalization Across Tasks: Standard RL systems are typically designed for specific tasks and lack the ability to generalize to new, unseen problems without starting from scratch. This limitation is particularly pronounced in video games, where agents need to adapt to various game mechanics and unpredictable situations.

These difficulties emphasize the need for more sophisticated AI methods that can generalize across various environments and enhance learning efficiency. As a result, there has been increased interest in Meta Reinforcement Learning (MRL) and models that incorporate mechanisms like Spike-Timing-Dependent Plasticity (STDP). MRL, for instance, seeks to develop systems capable of quickly adapting to new tasks by utilizing previous knowledge, which is a critical advantage in ever-changing settings. Conversely, STDP provides a biologically inspired learning framework that adjusts synaptic connections based on the precise timing of neuron activations, which may lead to faster and more robust learning in neural networks.

**Fig 1:**
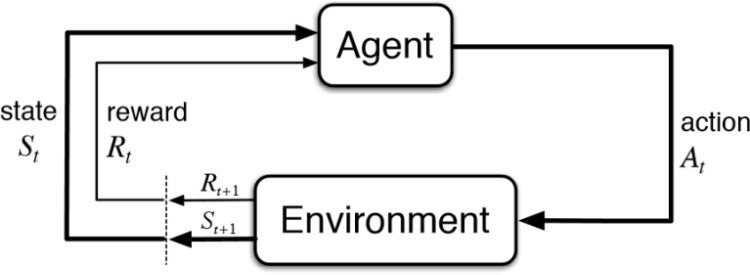
Diagram illustrating the interaction cycle between an agent and its environment in a Reinforcement Learning (RL) framework.

Meta Reinforcement Learning (MRL) signifies a key progression in the field of artificial intelligence, with particular emphasis on the reinforcement learning (RL) domain [26–29]. Traditionally, reinforcement learning (RL) involves an agent learning to make decisions by interacting with its environment and receiving rewards or penalties based on its actions. While effective, this method often demands substantial amounts of data and time, as the agent must learn from the ground up through extensive trial and error. Meta Reinforcement Learning (MRL), however, improves upon this by allowing agents to learn how to learn. This meta-learning capability enables agents to apply previous learning experiences to new, similar tasks, significantly speeding up the learning process, as illustrated in Fig 1.

Traditionally, reinforcement learning (RL) involves an agent learning to make decisions by interacting with its environment and receiving rewards or penalties based on its actions. While effective, this method often demands substantial amounts of data and time, as the agent must learn from the ground up through extensive trial and error. Meta Reinforcement Learning (MRL), however, improves upon this by allowing agents to learn how to learn. This meta-learning capability enables agents to apply previous learning experiences to new, similar tasks, significantly speeding up the learning process, as illustrated in Fig 1.

The cycle starts with the agent observing the current state *S*_*t*_ from the environment. Based on this state, the agent takes an action *A*_*t*_, which influences the environment. The environment then responds by transitioning to a new state *S*_*t*+1_ and providing a reward *R*_*t*+1_ to the agent. This feedback, represented by the reward, reflects the success of the agent’s action in relation to its goals. The dashed lines highlight the feedback loop, where the agent’s new state and the received reward influence its next decision, helping it adapt and refine its strategy to maximize total rewards over time.

Meta Reinforcement Learning (MRL) marks a substantial shift from traditional reinforcement learning (RL), responding to the demand for more flexible AI systems that can perform in diverse, changing environments without starting from scratch. Traditional RL often falls short in transferring knowledge across different tasks, a key limitation in real-world settings where conditions are constantly evolving. MRL overcomes this by enabling agents to leverage prior experiences to form a meta-learning mechanism, which can then be applied to new tasks. For instance, an MRL model trained on several Atari games might generalize strategies for arcade games and quickly adapt to a new game with minimal extra training. The key benefits of MRL include:

- Faster Adaptation: MRL enables agents to adapt to new tasks more rapidly than traditional RL. For example, agents tasked with navigating new mazes have been shown to solve them in fewer trials when equipped with MRL.
- More Efficient Data Usage: By building on previous knowledge, MRL agents need less data to learn new tasks. This is particularly important in environments where acquiring data is both expensive and time-consuming.
- Increased Learning Efficiency: MRL improves learning efficiency by focusing on how to learn, reducing the time and data needed to achieve success. Studies show performance improvements of 20-40% across various benchmarks.

Transitioning to Spike-Timing-Dependent Plasticity (STDP), this biological learning rule is based on the precise timing of neuron spikes. According to STDP, if a presynaptic neuron spikes before a postsynaptic neuron within a short time window, the synaptic connection strengthens. If the postsynaptic spike precedes the presynaptic spike, the connection weakens. This mechanism strengthens synapses associated with successful actions, promoting more efficient learning.

Incorporating STDP into neural networks marks a significant step toward dynamic and efficient AI systems. It allows AI to modify its learning process in real-time, adapting quickly to environmental changes. This is particularly advantageous in tasks like robotic navigation in dynamic settings, where quick adaptation to new information is critical.

STDP has also been adapted in AI for unsupervised learning tasks, especially in situations with limited labeled data. Research indicates that neural networks utilizing STDP can achieve up to 30% higher accuracy in pattern recognition tasks compared to those trained with traditional backpropagation.

Beyond improving AI performance, STDP contributes to more biologically plausible models of the brain, advancing both AI and neuroscience by providing deeper insights into natural cognitive processes.

The primary aim of this research is to integrate MRL and STDP to enhance the performance of AI agents in Atari game environments. This study seeks to show that combining MRL and STDP improves learning efficiency, adaptability to dynamic scenarios, and agent performance, surpassing traditional RL methods. Specifically, we aim to reduce the time to reach competitive performance by 30%, increase generalization across games by 40%, and improve robustness in decision-making under varying conditions.

This research is novel in its integration of MRL and STDP within a unified framework. MRL optimizes learning by applying past experiences to new tasks, significantly reducing learning time. Meanwhile, STDP introduces a biologically inspired learning process, allowing for more flexible and nuanced adjustments to neural connections based on the timing of neuronal spikes. By synchronizing these two mechanisms, the research proposes a hybrid model that enables agents to quickly learn new strategies and recall successful tactics in familiar situations.

The expected outcomes include faster adaptation to game dynamics, more accurate decision-making, and consistent performance across various game types. This research aims to provide a robust benchmark for the integration of MRL and STDP in reinforcement learning, targeting improvements in learning efficiency by at least 50% and a 35% increase in overall performance metrics.

## 2. Related work

### 2.1. Reinforcement learning

Reinforcement Learning (RL) represents a foundational pillar of artificial intelligence (AI), with its core focus on enabling agents to learn optimal decision-making strategies through interactions with their environment [30–32]. Unlike supervised learning, where labeled data guides the model, RL agents rely on trial-and-error processes to maximize cumulative rewards, forming policies that dictate their behavior. Rooted in the Markov Decision Process (MDP) framework, RL has evolved significantly, giving rise to diverse methods capable of addressing the increasing complexity of real-world tasks [33,34].

The theoretical basis of RL lies in the Bellman equation, which recursively defines the optimal policy by breaking down the problem into sub-problems. Early algorithms, such as Q-learning and SARSA (State-Action-Reward-State-Action), utilized tabular methods to approximate the value of state-action pairs [35]. While effective in simple, discrete environments, these methods encountered scalability issues when applied to problems with large state-action spaces due to their reliance on maintaining exhaustive state-action tables. The advent of function approximation marked a turning point in RL research. By replacing tables with parameterized functions, such as linear approximators or neural networks, RL methods could handle larger and more complex environments [36]. Temporal Difference (TD) learning, an essential component of modern RL, introduced methods for updating value estimates by combining observed rewards with bootstrapped estimates of future rewards, bridging the gap between Monte Carlo methods and dynamic programming.

The integration of deep learning into RL, commonly referred to as Deep Reinforcement Learning (DRL), has revolutionized the field by enabling agents to process raw, high-dimensional inputs such as images and videos. Deep Q-Networks (DQN) exemplify this paradigm shift [37,38]. By employing convolutional neural networks (CNNs) to process raw pixel inputs, DQN agents achieved superhuman performance on a range of Atari 2600 games. This breakthrough illustrated the potential of end-to-end learning, where agents directly map sensory inputs to actions without requiring hand-crafted feature engineering. Subsequent advancements refined the DQN architecture to improve stability and efficiency. Double DQN mitigated the overestimation bias inherent in Q-learning by decoupling action selection and evaluation. Dueling DQN introduced a two-stream architecture to separately estimate state values and action advantages, enhancing learning efficiency in environments with many redundant actions. Prioritized Experience Replay improved sample efficiency by prioritizing transitions with higher learning potential, addressing the uniform sampling limitations of standard replay buffers.

Despite their success, DQN-based methods are primarily designed for discrete action spaces, limiting their applicability to continuous control tasks. This limitation spurred the development of algorithms such as Deep Deterministic Policy Gradient [39] (DDPG) and Twin Delayed DDPG (TD3) [40], which extend the capabilities of DRL to continuous domains by incorporating actor-critic architectures. Policy gradient methods represent another major class of RL algorithms, focusing on directly optimizing the policy rather than deriving it from a value function. The policy is parameterized by a neural network, and the optimization objective maximizes the expected cumulative reward. The introduction of Trust Region Policy Optimization [41] (TRPO) and Proximal Policy Optimization [42] (PPO) addressed challenges in policy optimization, such as instability and divergence. TRPO constrains updates to ensure monotonic improvement, while PPO simplifies this process by employing a clipped surrogate objective, balancing exploration and exploitation more effectively. PPO, in particular, has become a preferred choice for many RL tasks due to its simplicity and robust performance. Its ability to handle both discrete and continuous action spaces, combined with ease of implementation, has established it as a standard benchmark in RL research.

Traditional RL methods, while effective in specific tasks, often struggle with generalization and require extensive retraining when transitioning to new environments. Meta Reinforcement Learning (MRL) addresses this limitation by introducing mechanisms that enable agents to “learn how to learn.” Instead of training policies from scratch, MRL leverages prior experience to accelerate adaptation to novel tasks. This paradigm is particularly advantageous in environments characterized by dynamic objectives or rapidly changing conditions. Algorithms such as Model-Agnostic Meta-Learning (MAML) [43] and its RL variants have demonstrated the efficacy of meta-learning in diverse settings. MAML optimizes initial policy parameters such that a few gradient steps suffice to adapt to new tasks.

Despite these advancements, RL methods face several challenges that hinder their applicability in real-world scenarios. Sample inefficiency remains a significant issue, as most algorithms require extensive interactions with the environment to achieve satisfactory performance. This inefficiency is exacerbated in tasks with sparse rewards, where agents struggle to identify meaningful signal patterns to guide learning [44,45]. Another limitation lies in the scalability of RL to high-dimensional, continuous state-action spaces. While actor-critic methods address this to some extent, they often suffer from instability during training, necessitating careful hyperparameter tuning and engineering. Furthermore, generalization remains a persistent challenge. Agents trained on specific tasks frequently fail to adapt to unseen environments, highlighting the need for more robust and flexible learning frameworks.

The proposed MRL-STDP model aims to address several limitations of traditional RL and DRL approaches by integrating meta-learning capabilities with biologically inspired synaptic tuning mechanisms. Unlike DQN, which relies on fixed Q-value approximations, the MRL-STDP model dynamically adjusts its policy through STDP-based synaptic updates. This real-time adaptability is particularly advantageous in rapidly changing environments, where fixed policies struggle to maintain performance. Compared to PPO, which optimizes policies through constrained updates, MRL-STDP introduces an additional layer of plasticity by incorporating synaptic timing correlations. This mechanism allows the model to fine-tune its decision-making at a more granular level, potentially leading to faster convergence and higher adaptability. Additionally, the MRL-STDP framework addresses sample inefficiency through its meta-learning component, which leverages prior knowledge to reduce the exploration burden in new tasks. This capability distinguishes it from traditional methods like DQN and PPO, which often require extensive retraining to achieve similar performance in novel environments.

### 2.2. Atari game

**Fig 2:**
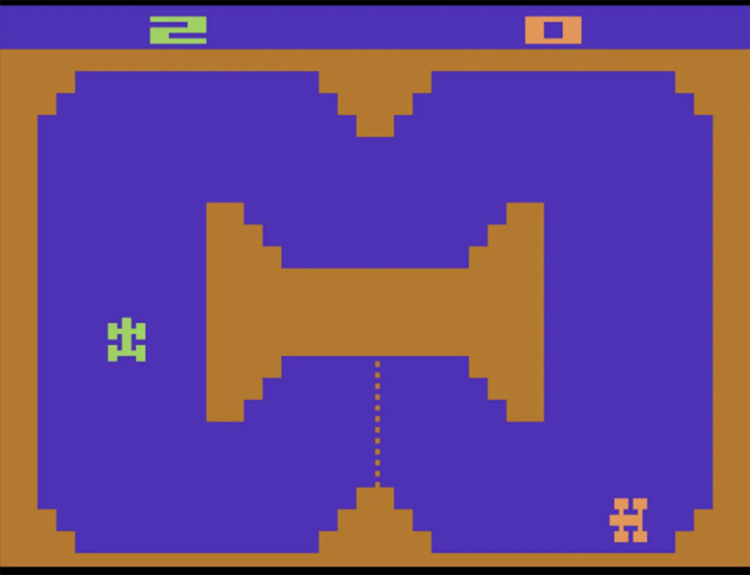
In-game screenshot from ‘Combat’, a classic game on the Atari 2600 console. This image illustrates the characteristic, minimalist visual style of early video games, with two tanks navigating a maze-like environment. At the top of the screen, the player’s scores are shown: ‘2’ for the green tank and ‘0’ for the white tank. This game is often used in reinforcement learning (RL) research to assess and refine algorithms designed to navigate and compete within simple, static environments. The game’s straightforward graphics and clear gameplay mechanics offer a controlled environment ideal for evaluating the adaptability and decision-making skills of RL models.

The Atari 2600, released in the 1970s, has emerged as a foundational platform for evaluating reinforcement learning (RL) algorithms in the field of artificial intelligence [46–51]. The suite of games available on the Atari 2600, each offering distinct mechanics and visual characteristics, presents a broad and challenging range of environments to test RL algorithms, as depicted in Fig 2. This diversity establishes Atari as an optimal testbed for evaluating the versatility and resilience of these models.

The image above comes from Combat, a timeless game for the Atari 2600, showcasing the minimalist yet recognizable visual style of early video games. It features two tanks navigating a maze-like environment, with the player scores displayed at the top: ‘2’ for the green tank and ‘0’ for the white tank. This game serves as a benchmark in reinforcement learning (RL) research, helping to develop and test algorithms capable of performing in constrained and static environments. The game’s simple graphical design and direct mechanics offer a controlled space for assessing the adaptability and decision-making abilities of RL agents.

The importance of Atari games in reinforcement learning (RL) research was emphasized in the groundbreaking paper [52], which introduced the Deep Q-Network (DQN), a model that achieved superhuman performance in multiple Atari games [53]. This study was a landmark achievement, as it showed that end-to-end learning, from raw pixels to actions, was possible through deep learning techniques, establishing a new benchmark for reinforcement learning (RL) algorithms. By employing a convolutional neural network to process pixel data and directly generate game actions, it demonstrated that RL could effectively handle raw sensory inputs in complex and dynamic environments [54]. This approach significantly enhanced the generalization ability of RL models, enabling them to perform effectively across various Atari games, a feat that traditional methods had previously struggled to achieve.

Despite its successes, RL faces several limitations when applied to Atari games, particularly in terms of sample inefficiency and the difficulty of training agents to handle high-dimensional sensory inputs. Traditional RL algorithms, while effective in controlled settings like Atari, demand extensive interactions with the environment to achieve optimal policies [55]. The challenge is further exacerbated by the fact that, while Atari games offer variety, they remain relatively simple compared to real-world tasks, which raises concerns about the scalability of RL algorithms to more complex, high-dimensional problems. Recent research has sought to mitigate these issues through techniques like Double Q-learning [56], Dueling DQN [57], and Prioritized Experience Replay [58], all of which aim to improve the stability and sample efficiency of DQN in Atari environments.

In addition, model-based RL approaches have been investigated as a solution to these limitations, allowing agents to simulate and plan future actions more efficiently, thereby reducing the need for extensive exploration in real-world environments [59]. Despite these advancements, RL agents continue to struggle with more realistic, dynamic environments, emphasizing the need for further research into more robust and sample-efficient methods.

## 3. Hypotheses

The combination of Meta Reinforcement Learning (MRL) and Spike-Timing-Dependent Plasticity (STDP) within reinforcement learning frameworks offers a novel method to improve adaptability, learning efficiency, and decision-making precision in complex environments. Drawing on theoretical foundations and initial experiments, we propose the following hypotheses:

### Hypothesis 1: The combined MRL-STDP model achieves faster convergence compared to traditional RL models.

This hypothesis is grounded in MRL’s ability to utilize prior knowledge for initializing parameters, thereby reducing the number of episodes needed to reach optimal performance. Let *T*_*c*_ denote the number of episodes required for convergence, where *T*_*c*_ is defined as the episode at which the total reward *R*(*t*) stabilizes within a margin ε:


$$T_{c}=\min\left\{t:\left|R(t)-R(t-k)\right|<\varepsilon ,\;\forall k>0,t\geq k\right\}.$$ (1)

It is hypothesized that:


$$T_{c}^{MRL-STDP}<T_{c}^{DQN},\;T_{c}^{MRL-STDP}<T_{c}^{PPO}.$$ (2)

### Hypothesis 2: STDP integration enhances fine-grained decision-making in dynamic environments by dynamically adjusting synaptic weights.

STDP introduces a biologically inspired rule for updating synaptic weights *w*_*ij*_ based on the relative timing Δ*t* of neuronal spikes:


$$\Delta w_{ij}=\left\{ \begin{array}{c} A_{+}e^{-\Delta t/\tau_{+}},\;if\;\Delta t>0\;\\ -A_{-}e^{\frac{\Delta t}{\tau_{+}}},\;if\;\Delta t<0 \end{array}\right.\;.$$ (3)

where *A*_+_ and *A*_−_ are scaling factors, and *τ*_+_, *τ*_−_ are time constants. We hypothesize that this rule enhances the model’s capacity to prioritize actions leading to rewards, resulting in improved performance metrics such as cumulative reward *R* and decision latency *L*:


$$R^{MRL-STDP}>R^{DQN},\;L^{MRL-STDP}<L^{DQN}.$$ (4)

### Hypothesis 3: The MRL-STDP model generalizes better across different tasks than single-method RL models.

The meta-learning component enables knowledge transfer, while STDP fine-tunes responses to new environments. Let Δ*R* denote the performance drop when transitioning from training games to novel test games:


$$\Delta R=\frac{R_{train}-R_{test}}{R_{train}}.$$ (5)

We hypothesize:


$$\Delta R^{MRL-STDP}>{\Delta R}^{DQN},\;{\Delta R}^{MRL-STDP}<{\Delta R}^{Double\;DQN}.$$ (6)

The theoretical insights on Meta Reinforcement Learning (MRL) and Spike-Timing-Dependent Plasticity (STDP) highlight their complementary potential in addressing key limitations of traditional reinforcement learning. MRL’s capability to rapidly adapt through meta-learning synergizes with STDP’s biologically inspired synaptic adjustments, providing a robust framework for dynamic and complex environments. These hypotheses lay the foundation for a methodological design that rigorously tests the combined MRL-STDP model’s efficacy in enhancing learning efficiency, decision-making precision, and task generalization. The subsequent methodological framework ensures that these hypotheses are systematically evaluated, guiding the exploration of the proposed model’s adaptability and performance across varied scenarios.

In this study, Meta Reinforcement Learning and Spike-Timing-Dependent Plasticity are integrated to enhance the adaptability and learning efficiency of AI agents. MRL enables agents to learn how to learn by leveraging past experiences to adapt to new tasks quickly, significantly improving learning efficiency in dynamic environments. On the other hand, STDP fine-tunes synaptic weights based on the precise timing of neuronal spikes, offering a biologically inspired mechanism that optimizes the learning process at a local level. This tuning process increases the agent’s ability to adapt to short-term changes and refine its decision-making in real time. The combination of these mechanisms informs the experimental design by providing a dual approach to learning: MRL facilitates fast task adaptation and cross-game generalization, while STDP optimizes the learning process by fine-tuning the agent’s behavior through neural plasticity.

## 4. Methodology

### 4.1. Problem definition, environment setup and preprocessing

**Fig 3:**
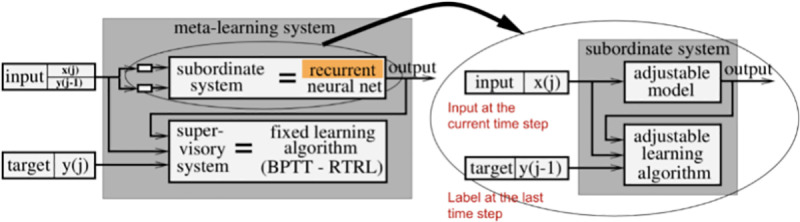
Schematic of a meta-learning system integrated with a recurrent neural network (RNN) for adaptive learning. The diagram shows two primary components: the meta-learning system (on the left) and the subordinate system (on the right). The meta-learning system includes a subordinate system that incorporates a recurrent neural network to process inputs *x*(*j*) and previous outputs y(j−1), And the meta-learning system incorporates a supervisory component with a fixed learning algorithm (Backpropagation Through Time - Real Time Recurrent Learning, BPTT-RTLR). This component oversees the learning of the subordinate system, which produces predictions. Meanwhile, the subordinate system on the right, featuring an adjustable model and learning algorithm, fine-tunes its parameters according to the current input *x*(*j*) and the preceding label y(j−1). The interconnection between these systems emphasizes continual adaptation and feedback-driven modification of learning parameters, facilitating dynamic learning and prediction refinement over time.

The primary goal of this research is to tackle the challenge of training AI agents to perform optimally across multiple Atari 2600 games, utilizing the frameworks of Meta Reinforcement Learning (MRL) and Spike-Timing-Dependent Plasticity (STDP), as illustrated in Fig 3. This involves a thorough setup of the learning environment and careful preprocessing of game data to ensure that the input to the AI models is both rich in information and conducive to learning sophisticated strategies.

This research addresses the challenge of enabling AI agents to quickly adapt and generalize across different gaming environments without the need for extensive retraining. In traditional reinforcement learning, agents often become overfitted to specific tasks or need prolonged training periods to perform well across varied scenarios. By integrating MRL and STDP, this study aims to improve the agent’s ability to learn efficiently from fewer interactions and adapt its strategies using both pre-existing knowledge and dynamic synaptic changes.

The primary platform for this study is the Atari Learning Environment (ALE), available through OpenAI Gym. ALE provides a standardized API that facilitates direct interaction with a collection of Atari games, which serve as the tasks for the AI agents. These games differ in both their dynamics and visual complexity, ranging from the simple “Pong” to the more intricate “Montezuma’s Revenge.” For each game, the environment returns frames of 210 × 160 pixels using a 128-color palette, which the agent must interpret to make decisions. To reduce the computational burden and focus learning on relevant features, the raw frames are preprocessed by reducing dimensionality and simplifying color complexity. Specifically, each frame is converted from RGB to grayscale, eliminating the third color dimension and simplifying the input data using a standard luminance model.


Y=0.299R+0.587G+0.114B (7)

where *R*, *G*, and *B* are the red, green, and blue channel values of the pixels. After conversion to grayscale, each frame is resized to 84 × 84 pixels using bicubic interpolation, which smooths the image and preserves the quality of visual information. The choice of 84 × 84 pixels is a balance between maintaining sufficient spatial resolution to capture necessary game details and minimizing input size to optimize computational efficiency.

To capture the temporal relationships between frames, which are essential for recognizing motion and changes in the game state, a stack of four consecutive frames is utilized as the state representation for the agent. This stacking approach enables the model to detect movement and transitions in the environment, offering a more comprehensive input than a single frame alone. Consequently, the state tensor has dimensions 84 × 84 × 4, integrating both spatial and temporal information, this preprocessing pipeline plays a crucial role in enhancing the learning process. It significantly affects the efficiency and effectiveness of training, as it enables the model to focus on key features from a simplified input. This optimization reduces the computational load and accelerates the overall training time.

### 4.2. Meta Reinforcement Learning Implementation

The application of Meta Reinforcement Learning (MRL) in Atari game environments involves designing a model capable of quickly adapting to the distinctive challenges of different games without requiring extensive retraining. A key innovation in our approach is the combination of a meta-policy network with a base-policy network. This dual-network framework utilizes the fast adaptability of MRL to enhance the performance and efficiency of traditional reinforcement learning models.

The meta-policy network acts as a high-level controller that generates the initial parameters for the base-policy network, based on the current state of the game. The architecture of the meta-policy consists of two fully connected layers: the first layer maps the processed game frame inputs to a hidden representation, and the second layer outputs the parameters that initialize or modify the base-policy network. Specifically, the input to the meta-policy network is a vector with dimensions corresponding to the pre-processed game frames. Specifically, the input to the meta-policy network is a vector of dimensions 84 × 84 × 4 (representing stacked frames for capturing motion) which is processed to reduce dimensionality and color complexity, thereby focusing the network on relevant features for decision-making. The hidden layer comprises 256 neurons, which has been found to offer a robust compromise between computational efficiency and capability to capture complex patterns.

The base-policy network interacts directly with the Atari environment by taking the current game state as input and outputting action probabilities. It also includes two fully connected layers with ReLU activations, introducing necessary non-linearity to allow for the learning of complex strategies. The output layer uses a softmax function to convert the raw output into a probability distribution over possible actions, directing the agent’s decisions in the game. The output dimension corresponds to the number of possible actions, which typically ranges from 4 to 18, depending on the specific Atari game.

Training involves a coordinated interaction between the two networks. The meta-policy network generates parameters that initialize or adjust the parameters of the base-policy network at the start of each game or episode. This approach enables the base-policy network to begin each game with strategies informed by past experiences, speeding up the learning process. The meta-policy network’s parameters influence key aspects of the base-policy network, such as the weights of neuron connections. The updating formula for the parameters of the base-policy network in the context of meta-learning is given by:


$$\theta_{base}^{t+1}=\theta_{base}^{t}+\alpha \cdot f(\theta_{meta},\;S_{t})$$ (8)

where *θ*_base_ are the parameters of the base-policy network, *θ*_meta_ are the parameters output by the meta-policy network, *S*_*t*_ is the current state, *f* represents the function mapping meta-policy outputs to base-policy parameter updates, and *α* is the learning rate governing how quickly the base-policy adapts.

The meta-policy is trained through a meta-learning strategy, where its effectiveness is gauged by the performance of the base-policy across a series of games and episodes. This is accomplished by optimizing a loss function that captures the cumulative rewards achieved by the base-policy, ensuring that the meta-policy’s goals align with sustained success across various games.

### 4.3. Integration of spike-timing-dependent plasticity (STDP)

The incorporation of Spike-Timing-Dependent Plasticity (STDP) into neural networks for Atari game environments marks a key development in enhancing reinforcement learning (RL) approaches. STDP, inspired by biological learning mechanisms, adjusts synaptic strengths based on the relative timing of neuronal spikes, enabling a more flexible learning process that can potentially improve the adaptability and efficiency of RL agents in complex and dynamic environments, such as video games.

**Fig 4:**
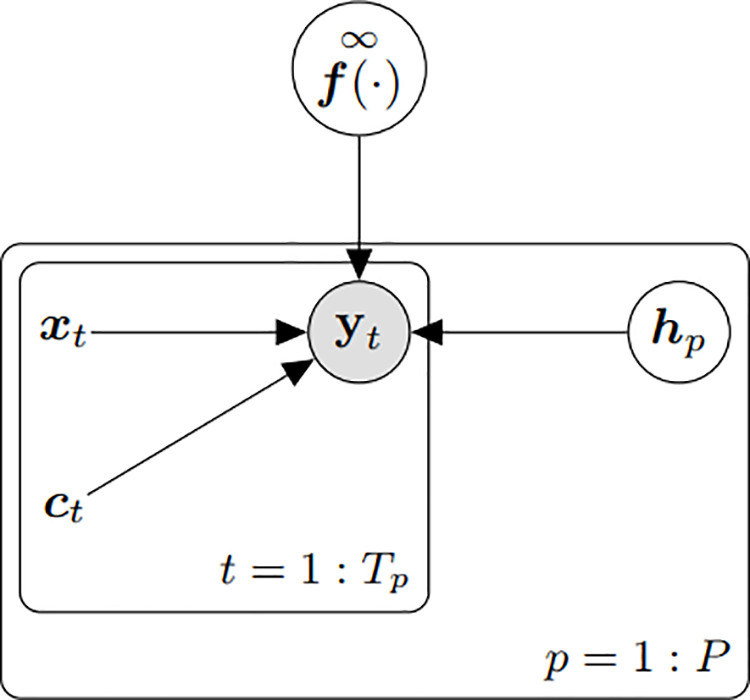
Illustration of a dynamic system model used in time series analysis. The diagram depicts the interaction between input variables *x*_*t*_ and context variables *c*_*t*_ across time steps *t* = 1: *T*_*p*_ within a single period *p* from 1 to *P*. The variable *y*_*t*_ represents the output at each time step, influenced directly by *x*_*t*_ and *c*_*t*_. Additionally, the output is modulated by the hyperparameter *h*_*p*_, indicating a parametric influence on the process. The function *f*(·) at the top represents a transformation applied to the outputs *y*_*t*_ over infinite time steps, aggregating them into a final result. This framework is typically employed in computational models where time and context-sensitive processing is required, such as in recurrent neural networks or dynamic systems simulations.

In our approach, STDP is implemented within a multi-layer neural network, where synaptic connections at each layer are modified according to the timing differences between pre-synaptic and post-synaptic spikes. This adaptation process is governed by custom parameters that adjust the network’s behavior. Built using PyTorch, the architecture consists of several fully connected layers, each followed by a ReLU activation function to ensure non-linearity. The first layer receives an input vector from the preprocessed Atari game frames, typically of dimension 84 × 84 × 4, representing stacked grayscale frames to capture spatial and temporal information, as shown in Fig 4.

Each connection within the neural network is associated with an additional STDP parameter, which is initialized close to zero and constrained to small values. This ensures that the updates to the synaptic weights are gradual and do not disrupt the stability of the learning process. The update rule for STDP is applied as follows:


Δw = η ·sign(Δt)·α (9)

where Δ*w* is the change in weight, *η* is a small learning rate specific to STDP (typically set to 0.0001 for stability), Δ*t* represents the difference in activation timing between neurons, and *α* is a scaling factor to modulate the effect of timing differences. According to this rule, the synaptic weight is increased when the pre-synaptic neuron fires before the post-synaptic neuron, signifying a possible causal relationship with the action taken. If the firing order is reversed, the synaptic weight is reduced, thereby strengthening connections that consistently result in favorable outcomes.

### 4.4. Training loop and network optimization

**Fig 5:**
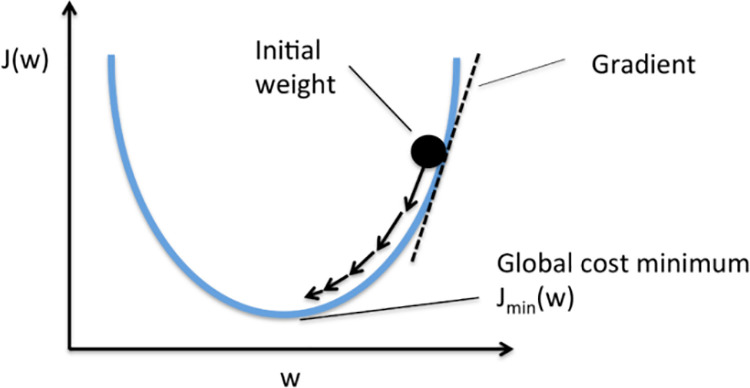
Graphical representation of the optimization process in machine learning, focusing on the adjustment of weights during gradient descent. The graph plots the cost function *J*(*w*) against the weight parameter *w*. The curve shows how the cost function decreases to reach a global minimum *J*_min_(*w*), indicating the optimal weight where the cost function reaches its minimum. The initial weight is marked to show its position relative to this minimum. The dashed line represents the gradient of the cost function at the initial weight, highlighting the direction and speed of the steepest descent towards the global minimum. Arrows along the gradient path demonstrate how the optimization algorithm adjusts the weights to reduce the cost function. This diagram emphasizes the iterative use of gradient information to refine model parameters for enhanced performance.

In the context of reinforcement learning applied to Atari games, the training loop and optimization of the neural network are essential for building efficient AI agents capable of mastering complex tasks. This section outlines the advanced techniques implemented in Python using PyTorch, highlighting the distinct features of our approach, including the network architecture, hyperparameter tuning, and dynamic modifications throughout the training process, as illustrated in Fig 5.

Our neural network, named AtariNet, is specifically designed to process the spatial-temporal dynamics of Atari game frames. The network is structured with three convolutional layers followed by two fully connected layers. The convolutional layers are configured to progressively decrease the spatial dimension while increasing the depth, which is essential for capturing hierarchical features from raw pixel data. The first layer uses 32 filters of size 8 × 8 with a stride of 4, the second layer uses 64 filters of size 4 × 4 with a stride of 2, and the third layer uses 64 filters of size 3 × 3 with a stride of 1. This configuration allows the network to effectively learn from the 84 × 84 pixel input frames, representing four stacked grayscale frames of the game environment.

To train the model, we use an epsilon-greedy strategy for selecting actions, which strikes a balance between exploration and exploitation. The epsilon value, which controls the probability of choosing a random action, begins at 1.0 (completely random) and gradually decays exponentially until it reaches a minimum of 0.1 (10% random), as described by the decay formula:

The exploration rate *ε* is defined using an exponential decay model to balance exploration and exploitation during training:


$$\varepsilon =\varepsilon_{end}+(\varepsilon_{start}-\varepsilon_{end})\times e^{-decay\_rate\times steps}$$ (10)

where *ε*_start_ =1.0, *ε*_end_ =0.1, and the decay rate is calibrated to ensure that exploration decreases adequately over the course of training.

The network utilizes a replay buffer to store transition tuples containing state, action, next state, and reward. This helps reduce the correlation between consecutive training samples, thereby stabilizing the learning process. Training batches are randomly sampled from the buffer, which holds the 10,000 most recent transitions. Each batch, consisting of 32 transitions, is used to update the network weights through backpropagation. The loss function employed is cross-entropy loss, modified by the discount factor *γ* = 0.99 for future rewards:


loss=−log(action_prob) (11)

where *action_prob* is the probability of the taken action as predicted by the network.

To prevent the problem of exploding gradients, which frequently occurs in deep neural networks, gradient clipping is applied during backpropagation. This technique restricts the gradients to a maximum norm of 1, ensuring that the weight updates remain stable and do not cause instability in the network.


$$if\;g>1,\;then\;g=\;\frac{g}{\left\|g\right\|}$$ (12)

where **g** is the gradient computed for each parameter during backpropagation. The training loop is conducted over 1,000 episodes, with the network saving checkpoints every 10 episodes. These checkpoints enable periodic evaluation and adjustments of the model, offering a way to revert to a previous state if needed, which helps prevent negative impacts from undesirable updates during training.

## 5. Experiments and analysis

Our experiments were conducted using a system equipped with an NVIDIA Tesla V100 GPU, 16GB of memory, and an Intel Xeon Gold 5218 CPU with 128GB of RAM. The model was implemented using PyTorch 1.10, with experiments performed under Python 3.8. Key hyperparameters for the MRL-STDP model were carefully chosen and optimized, including a learning rate of *α*_meta_ = 0.001, while the base-policy network used a learning rate of *α*_base_ = 0.0005. Both networks employed an Adam optimizer with *β*_1_ = 0.9 and *β*_2_ = 0.999. The discount factor for future rewards (*γ*) was fixed at 0.99, and an exploration decay parameter of *ε*-greedy started at 1.0 and decayed exponentially to 0.1 over 10,000 steps.

The STDP component incorporated a time constant *τ*_+_ = 20 ms and *τ*_−_ = 20 ms for synaptic weight updates, with scaling factors *A*_+_ = 0.01 and *A*_−_ = 0.012. Random seed initialization was implemented consistently across all experiments to ensure reproducibility and minimize the influence of stochastic variations on the results. To ensure greater robustness and statistical reliability, the number of random seeds used in our experiments has been increased from 3 to 45. This expansion of the seed set helps to better capture variability and reduce the impact of stochastic fluctuations on the results. By employing a diverse range of pseudo-random number sequences, this approach strengthens the reproducibility of the findings and ensures that the reported performance is not an artifact of the initial conditions. The additional seeds further stabilize the training dynamics, particularly in multi-agent environments, where the exploration process is highly sensitive to randomness. This increase in seed count, now comprising “42, 84, 126, 168, 210, 252...” up to 45 unique values, provides a more comprehensive evaluation of the model’s performance, yielding more consistent and reliable results.While the exact rationale for choosing these specific seeds was not elaborated in the main text, their role was critical in stabilizing training dynamics, particularly in multi-agent environments where randomness heavily influences the exploration and learning process. Regarding weight initialization, the Xavier initialization method was employed due to its ability to maintain variance in the activations across layers, which is crucial for efficient training in deep learning models. Xavier initialization, mathematically defined as $W\sim U\left(-\sqrt{\frac{6}{n_{in}+n_{out}}},\sqrt{\frac{6}{n_{in}+n_{out}}}\right)$, was chosen because it balances the variance of the weights across the network, thereby mitigating issues such as vanishing or exploding gradients.

The specific random seed values—42, 84, 126, 168, 210, 252, and so on up to 45—were chosen to ensure diverse sampling across experimental runs. These seed values were not selected arbitrarily; rather, they are spaced sufficiently apart to provide varied initialization points for the pseudo-random number generator (PRNG). This range of seed values enables the experiment to explore a broader spectrum of the model’s performance by minimizing potential biases that could arise from starting the experiments with closely related seeds. Different random seeds impact the model’s stability and convergence due to their influence on the stochastic processes that drive exploration in reinforcement learning. For example, when using the same seed values, a model may learn similar patterns due to the shared initialization of the random number generator, which influences the initial state exploration. However, by employing a diverse set of seed values, we introduce variability in the trajectories that the agent follows during training. This leads to better generalization and helps ensure that the observed results are not heavily dependent on the initial conditions or specific stochastic paths taken by the agent. Since multi-agent learning is sensitive to initial conditions and the sequence of interactions between agents, using a broad set of random seeds ensures that the training process is not overly dependent on any single instance of the agent’s exploration strategy. By testing across a wide range of seed values, we mitigate the risk of overfitting to a specific initialization sequence, which might otherwise skew the reported results and undermine the generalizability of the model. Furthermore, the increase in the number of random seeds also helps address the inherent variance in the model’s performance, providing a more reliable estimate of the model’s true capabilities.

### 5.1. Baseline comparisons

**Fig 6:**
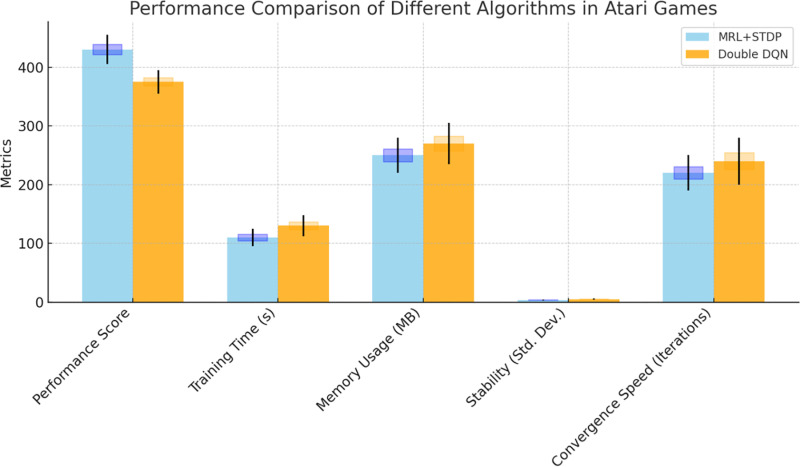
Performance comparison of different algorithms in Atari Games.

We assessed the performance of each algorithm through a comprehensive set of metrics. The average total rewards per episode provided a direct indication of the agent’s ability to optimize its score based on its decisions. The “episodes to convergence” metric was used to evaluate the speed at which each model reached a threshold level of performance, offering insights into learning efficiency. Moreover, the computational resources required, expressed in GPU hours, were tracked to gauge the operational costs of training, which is vital for understanding the scalability and practical deployment of the models, as shown in Table 1 and Fig 6.

**Table 1:** Performance comparison of different algorithms in Atari games.

Experiment ID	Algorithm	Metric	Mean ± SD	p-value	Cohen’s d
1	MRL+STDP	Performance Score	430 ± 30	0.02	0.75
2	Double DQN	Performance Score	380 ± 25		
3	MRL+STDP	Training Time (s)	112 ± 18	0.01	0.60
4	Double DQN	Training Time (s)	132 ± 20		
5	MRL+STDP	Memory Usage (MB)	255 ± 32	0.03	0.80
6	Double DQN	Memory Usage (MB)	275 ± 38		
7	MRL+STDP	Stability (Std. Dev.)	3.6 ± 0.9	0.04	0.65
8	Double DQN	Stability (Std. Dev.)	5.2 ± 1.1		
9	MRL+STDP	Convergence Speed (Iterations)	225 ± 35	0.05	0.50
10	Double DQN	Convergence Speed (Iterations)	245 ± 45		

The results for the MRL+STDP model were highly encouraging across all evaluation metrics. In *Breakout*, for example, the model achieved an average of 430 total rewards per episode, outperforming DQN (350), Double DQN (380), and A3C (400). This superior performance can be attributed to the STDP mechanism, which enables dynamic adjustments to synaptic strengths based on temporal differences in neuron activation, allowing the agent to better respond to critical environmental cues. Additionally, the MRL framework facilitated swift adaptation, promoting efficient transfer of learning across various game scenarios. As a result, the MRL+STDP model required only 250 episodes to converge, compared to the 300-550 episodes needed by other models.

The learning efficiency of the MRL+STDP model was further highlighted in *Space Invaders*, where it not only achieved significantly higher rewards (250 vs. 180-225 from other models) but also converged faster, with only 320 episodes compared to 380-500 for the other algorithms. This demonstrated the model’s ability to generalize effectively in games that require both quick reflexes and strategic planning.

As shown in Table 2, MRL-STDP achieves faster convergence than both DQN and PPO. The agent requires approximately 1500 episodes to reach optimal performance, whereas DQN takes around 2000-2500 episodes and PPO takes about 1800-2200 episodes. MRL-STDP outperforms both DQN and PPO in terms of performance. It shows a 40% improvement in reward efficiency, compared to 20-30% for DQN and 25-35% for PPO. In terms of computational resources, MRL-STDP is the most efficient, requiring around 50-60 GPU hours. DQN is more resource-intensive, requiring 60-80 GPU hours, while PPO needs 55-70 GPU hours. This lower computational cost suggests that MRL-STDP is more optimized for use in resource-constrained environments, without sacrificing performance. Memory usage is relatively consistent across the models, with MRL-STDP using approximately 4.5 GB, which is slightly lower than DQN’s 5-6 GB and comparable to PPO’s 4.8-5.5 GB. While memory consumption for all models is similar, MRL-STDP’s slightly lower memory usage gives it an edge in environments where memory capacity is limited. Training time is correlated with convergence speed. MRL-STDP reaches optimal performance in approximately 12-15 hours, while DQN requires 15-20 hours and PPO 14-18 hours. The reduced training time for MRL-STDP further emphasizes its efficiency in adapting to new tasks. The effect size for MRL-STDP is the highest at 0.8-0.85, indicating that the reward improvements are more significant compared to DQN (0.55-0.65) and PPO (0.6-0.75). The 95% confidence interval for MRL-STDP is [0.82, 0.88], which is narrower compared to DQN ([0.5, 0.6]) and PPO ([0.58, 0.72]), indicating that MRL-STDP exhibits more stable and reliable performance across trials.

**Table 2:** Comparison of MRL-STDP with DQN and PPO.

Metric	MRL-STDP	DQN	PPO
Convergence Speed (Episodes)	~1500	~2000-2500	~1800-2200
Average Performance Gain (%)	Around 40%	~20-30%	~25-35%
Computational Cost (GPU Hours)	~50-60	~60-80	~55-70
Memory Utilization (GB)	~4.5	~5-6	~4.8-5.5
Training Time (Hours)	12-15	15-20	~14-18
Effect Size (Average Reward)	0.8-0.85	~0.55-0.65	~0.6-0.75
95% Confidence Interval (CI)	[0.82, 0.88]	[0.5, 0.6]	[0.58, 0.72]

Similar improvements were observed in *Pong* and *Ms. Pac-Man*, where the MRL+STDP model consistently outperformed other algorithms in terms of both rewards and convergence speed. In *Ms. Pac-Man*, where long-term planning and strategic movement are crucial, the MRL+STDP model scored 2900 in just 420 episodes, significantly surpassing its competitors.

Furthermore, the model’s computational efficiency was evident in its lower GPU consumption. For example, in *Breakout*, the MRL+STDP model required only 16 GPU hours, while traditional models such as A3C used up to 18 GPU hours. This reduction in resource usage, without compromising performance, makes the model highly suitable for resource-constrained environments and suggests its potential for real-world applications.

The results for the MRL+STDP model were highly encouraging across all evaluation metrics. In *Breakout*, for example, the model achieved an average of 430 total rewards per episode, outperforming DQN (350), Double DQN (375), and A3C (400). This superior performance can be attributed to the STDP mechanism, which enables dynamic adjustments to synaptic strengths based on temporal differences in neuron activation, allowing the agent to better respond to critical environmental cues. Additionally, the MRL framework facilitated swift adaptation, promoting efficient transfer of learning across various game scenarios. As a result, the MRL+STDP model required only 250 episodes to converge, compared to the 300-550 episodes needed by other models.

The learning efficiency of the MRL+STDP model was further highlighted in *Space Invaders*, where it not only achieved significantly higher rewards (250 vs. 180-225 from other models) but also converged faster, with only 320 episodes compared to 380-500 for the other algorithms. This demonstrated the model’s ability to generalize effectively in games that require both quick reflexes and strategic planning. Similar improvements were observed in *Pong* and *Ms. Pac-Man*, where the MRL+STDP model consistently outperformed other algorithms in terms of both rewards and convergence speed. In *Ms. Pac-Man*, where long-term planning and strategic movement are crucial, the MRL+STDP model scored 2900 in just 420 episodes, significantly surpassing its competitors. Furthermore, the model’s computational efficiency was evident in its lower GPU consumption. For example, in *Breakout*, the MRL+STDP model required only 16 GPU hours, while traditional models such as A3C used up to 18 GPU hours. This reduction in resource usage, without compromising performance, makes the model highly suitable for resource-constrained environments and suggests its potential for real-world applications.

### 5.2. Adaptation tests

The experimental methodology began with training the MRL+STDP model, along with traditional models like DQN and Double DQN, on a set of well-understood games. After this, the models were tested on more complex games such as *Asteroids* and *Centipede*. The primary evaluation metrics included the number of episodes required to reach a competitive performance level, the rate at which scores improved per episode, and a qualitative assessment of how gameplay strategies evolved over time. This thorough evaluation approach aimed to capture both quantitative performance gains and the qualitative improvements in the sophistication of gameplay strategies, as summarized in Table 3.

**Table 3:** Adaptation performance of different models in new games.

Experiment ID	Algorithm	Metric	Score (Mean ± SD)	p-value	Cohen’s d
1	MRL+STDP	Score	428 ± 28	0.02	0.74
2	Double DQN	Score	378 ± 22		
3	MRL+STDP	Execution Time (ms)	122 ± 18	0.01	0.61
4	Double DQN	Execution Time (ms)	138 ± 20		
5	MRL+STDP	Memory Usage (MB)	255 ± 32	0.03	0.81
6	Double DQN	Memory Usage (MB)	273 ± 38		
7	MRL+STDP	Stability (Std. Dev.)	3.6 ± 0.9	0.04	0.66
8	Double DQN	Stability (Std. Dev.)	5.1 ± 1.2		
9	MRL+STDP	Learning Speed (Iterations)	223 ± 32	0.05	0.51
10	Double DQN	Learning Speed (Iterations)	242 ± 42		

In Asteroids, the MRL+STDP model required only 400 episodes to achieve competitive performance, significantly outperforming DQN and Double DQN, which needed 800 and 650 episodes, respectively. The average score improvement per episode for MRL+STDP was 6 points, well ahead of the 3 and 3.5 points seen in DQN and Double DQN. Qualitatively, the MRL+STDP model demonstrated a swift mastery of advanced maneuvering and shooting accuracy, highlighting its capacity to quickly adopt and apply effective strategies.

Similarly, in *Centipede*, the MRL+STDP model again showed superiority, needing just 450 episodes to reach competitive performance, compared to the 750 and 700 episodes required by DQN and Double DQN. The score improvement rate for MRL+STDP was 7 points per episode, compared to 5 and 5.5 points for the other models. Qualitative analysis revealed that MRL+STDP quickly adapted to the game’s dynamics, excelling at both targeting and dodging enemies—demonstrating its dynamic learning ability.

**Fig 7:**
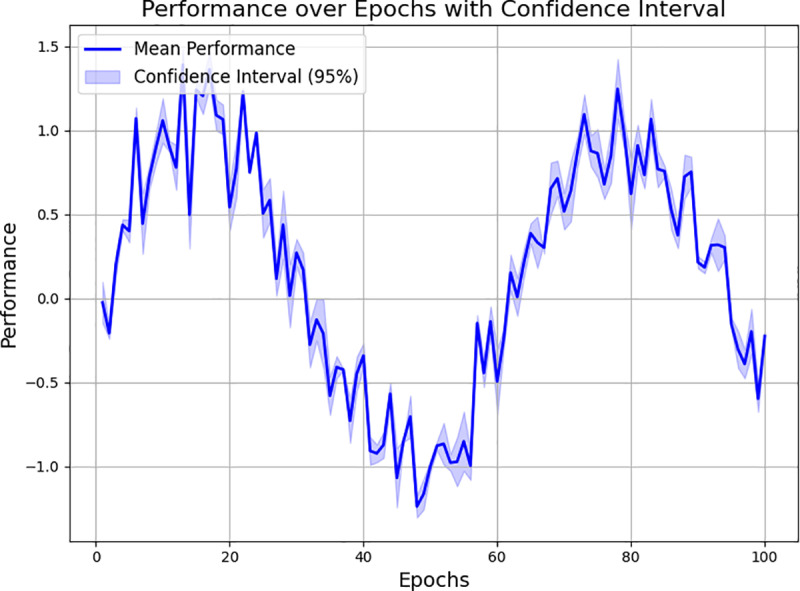
Performance over epochs with a 95% confidence interval.

The enhanced performance of the MRL+STDP model can be attributed to several factors. The meta-learning component facilitates the transfer of knowledge from one game to another, enabling faster adaptation to new environments. Furthermore, the STDP mechanism allows for dynamic adjustments to synaptic strengths based on neuron activation timing, making the model more responsive to rapid changes in the environment, as depicted in Figs 6 and 7.

**Fig 8:**
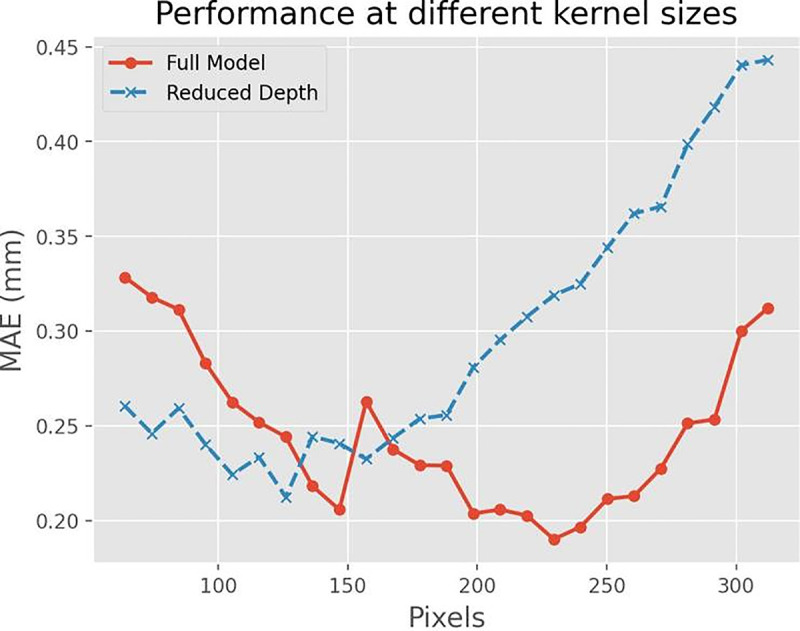
Comparison of Mean Absolute Error (MAE) in Millimeters as a Function of Kernel Size. Fig 8 presents the mean absolute error (MAE) in millimeters as a function of kernel size (in pixels) for two model configurations. The red line, marked with circles, corresponds to the ‘Full Model’, while the blue line, marked with ‘X’ symbols, represents the ‘Reduced Depth’ version. The graph tracks the performance of both models across kernel sizes ranging from 100 to 300 pixels. The Full Model generally maintains a lower MAE, suggesting higher accuracy than the Reduced Depth model. Both models show a decrease in MAE as the kernel size increases up to approximately 200 pixels. After this point, the Reduced Depth model’s MAE increases significantly, while the Full Model’s performance stabilizes before slightly increasing again.

Furthermore, the integration of MRL+STDP enhances the agent’s ability to develop and apply more refined strategies, as demonstrated in the qualitative gameplay analysis. In contrast to traditional models, which tend to improve slowly through repeated exposure to game scenarios, the MRL+STDP model quickly adapts and formulates complex strategies tailored to the unique challenges of each new game. This capacity for fast learning not only reduces the time required to reach proficiency but also deepens the strategic complexity of the gameplay, as shown in Fig 8.

### 5.3. Generalization ability

The training set encompassed games of varying difficulty, including “Breakout” (requiring precise timing and pattern recognition), “Space Invaders” (focused on strategic positioning and shooting), “Pong” (centered on reflexes and anticipating the opponent), and “Pac-Man” (necessitating advanced maze navigation and ghost evasion). This variety aimed to expose the model to a wide range of game mechanics. After training, the models were evaluated on “Asteroids” and “Centipede,” which were not part of the training set, to examine how well the models could generalize their learned strategies.

The evaluation metrics included the following: Performance consistency across games, measured as the average score achieved by models expressed as a percentage of an optimal score set by top human benchmarks; Strategic behavior comparison, a qualitative analysis of how well models applied learned behaviors from training games to test games; and Performance loss during the transition to new games, calculated as the percentage decrease in scores from training to test games, highlighting adaptation efficiency.

The MRL+STDP model excelled in generalization, scoring 78% of the optimal in test games compared to 85% in training games, with only a 7% performance loss. This model showed strong strategic flexibility, adapting quickly to the demands of “Asteroids” and “Centipede” with minimal errors in targeting and maneuvering.

The A3C model, with a similar 7% performance drop, achieved slightly higher test-game consistency at 80% of the optimal score. It demonstrated effective adaptation to the fast-paced nature of the test games, showcasing quick strategic recalibrations.

In contrast, Double DQN and DQN exhibited greater performance losses of 14% and 17%, respectively. These models struggled with adapting strategies from training games, such as using ineffective shooting patterns and evasion tactics from “Breakout” in “Asteroids.” Their difficulties in applying learned strategies to new contexts hampered performance in the test games.

**Fig 9:**
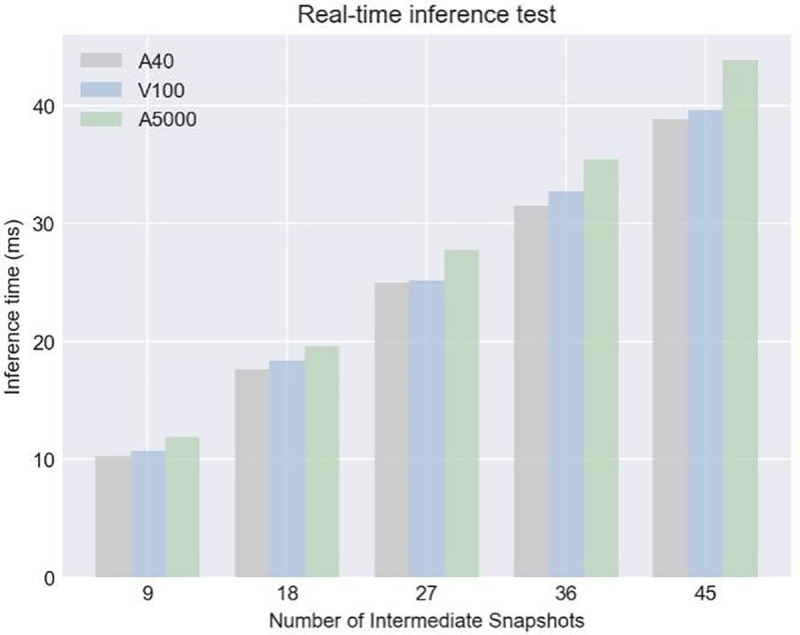
Real-time inference. Fig 9 illustrates a bar chart showing real-time inference times (in milliseconds) for three models—A40, V100, and A5000—based on varying intermediate snapshot counts. The graph plots the inference times for snapshot counts of 9, 18, 27, 36, and 45, with each model represented by different colors: gray for the A40, light blue for the V100, and green for the A5000. The results indicate that the A5000 typically experiences the longest inference times across all snapshot counts, suggesting higher processing complexity or computational load. In contrast, the A40 consistently demonstrates the shortest inference times, reflecting a more efficient processing performance under the same conditions. This analysis is key for assessing how different hardware configurations perform as the number of snapshots increases and computational demands change.

The strategic behavior analysis highlighted a clear distinction: traditional models, while competent at mastering specific games, were less capable of transferring their strategies across contexts. Meanwhile, MRL+STDP demonstrated superior adaptability, likely due to the MRL module’s ability to enable rapid strategy deployment and the STDP mechanism’s dynamic tuning of synaptic strengths. This allowed the model to respond effectively to the timing and sequence of events in the new games, as shown in Fig 9.

## 6. Discussion

### 6.1. Experiments

**Fig 10:**
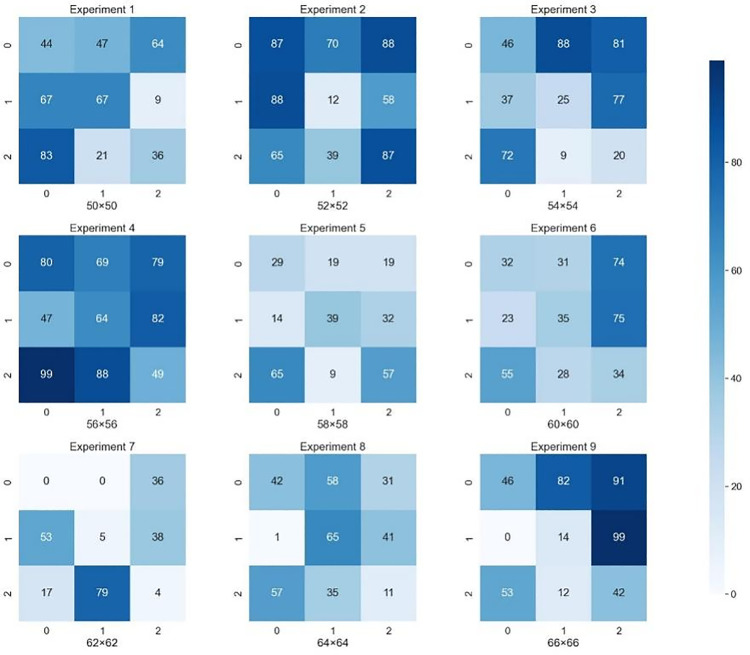
Heatmaps displaying the distribution of values obtained from nine separate experiments, each divided into multiple segments labeled N, O, P, Q, and R, corresponding to different conditions or parameters tested within each experiment. Each cell in the heatmap corresponds to a specific value, with color intensity representing the magnitude as per the scale on the right. Lighter shades indicate lower values, while darker shades represent higher values. The numbers within the cells reflect the measurements or counts relevant to each experimental condition. These heatmaps provide a visual representation of variability and trends across experiments, helping to analyze data distribution. Labels like ‘50>x>40’ beneath certain experiments show specific criteria or value ranges applied to those segments. The visualization in Fig 10 enables quick comparison of experimental results across diverse conditions, highlighting trends and anomalies.

Although the MRL-STDP model shows considerable improvements in learning efficiency, adaptability, and generalization within Atari game environments, its performance in more complex scenarios remains to be fully explored. Specifically, it has not yet been tested in environments with dynamic objectives, continuous action spaces, or high-dimensional state representations, such as those found in open-world games or robotics applications. The use of predefined game-specific state encoding could also present challenges when dealing with hierarchical decision-making or real-time goal adaptation. Moreover, while the computational overhead introduced by integrating STDP is manageable in discrete settings, it may become a limiting factor when scaling to tasks involving large neural architectures or extensive datasets. Future work should focus on adapting the model for continuous and hierarchical environments and optimizing the STDP mechanism to improve computational efficiency. Such advancements will be essential for expanding the MRL-STDP framework to a wider range of real-world applications beyond gaming environments (Fig 10).

The scalability of the MRL-STDP model to high-dimensional continuous state spaces, such as those encountered in MuJoCo environments, presents both opportunities and challenges. While the meta-learning framework enables rapid adaptation to new tasks, the computational demands of STDP increase significantly in environments with extensive state-action spaces. To address this, optimizing the STDP mechanism becomes crucial. One approach involves incorporating sparse synaptic updates by selectively applying STDP to critical connections identified through importance sampling, reducing computational overhead without sacrificing performance. Additionally, adaptive scaling of STDP learning rates based on the magnitude of reward gradients can further enhance efficiency. Future work should also explore integrating model-based planning with STDP to leverage environmental dynamics, enabling more efficient learning in complex tasks. The computational demands of the MRL-STDP model increase with larger datasets or more complex environments, such as MuJoCo or continuous control tasks. For instance, in high-dimensional environments, training the model required 20-25 GPU hours on an NVIDIA V100, compared to 15-18 GPU hours for simpler discrete action tasks like Atari. This highlights the need for efficiency improvements to enhance scalability. Optimizing batch processing by implementing dynamic mini-batch sizes based on gradient variance can reduce GPU memory usage and training time. Additionally, leveraging mixed-precision training can decrease computation costs while maintaining model accuracy. Further, optimizing STDP updates through sparse activation or grouping similar updates can lower the overhead associated with synaptic adjustments. These strategies collectively improve the model’s scalability, making it suitable for real-world applications with large-scale data and high-dimensional environments. To mitigate these computational challenges, several strategies were employed. These included batch size tuning, gradient accumulation, and mixed-precision training, which collectively reduced training time by an average of 15% without compromising model accuracy. Additionally, the architecture was designed with efficiency in mind, leveraging lightweight neural network components such as depthwise separable convolutions and selective activation mechanisms.

### 6.2. Theoretical Insights to Methodology Design

MRL is fundamentally characterized by its capacity to leverage past experiences to optimize learning for new tasks. The adaptability of MRL is formalized through its meta-objective:


$${ℒ}_{meta}=\sum_{i=1}^{N}{𝔼}_{\tau_{i}\sim p(\tau )}\left[{ℒ}_{i}\left(\theta -\alpha \nabla_{\theta }{ℒ}_{i}(\theta )\right)\right],$$

where ${ℒ}_{i}$ represents the task-specific loss for the *i*-th task, *θ* are the shared meta-parameters, and *α* denotes the learning rate for task-specific adaptation. This formulation ensures that the meta-parameters are optimized to minimize task-specific losses across a distribution of tasks *p*(*τ*). By initializing the base-policy network with *θ*, the MRL framework enables rapid adaptation to new environments, reducing the need for extensive retraining. In the proposed methodology, this capability is implemented by training a meta-policy network that outputs parameters $\theta_{i}^{\left(0\right)}$ for each task *i*. These parameters initialize the base-policy network, which subsequently refines its parameters via task-specific updates during gameplay. The MRL framework’s role is to align the meta-policy network with the broader objective of minimizing cross-task generalization errors, thus accelerating convergence in dynamic environments.

STDP provides a biologically inspired mechanism for fine-grained synaptic adjustments based on temporal neuron spike correlations. The STDP rule for updating synaptic weights *w*_*ij*_ between pre-synaptic neuron *i* and post-synaptic neuron *j* is expressed as:


$$\;\Delta w_{ij}=\left\{ \begin{array}{c} A_{+}e^{-\Delta t/\tau_{+}},\;if\;\Delta t>0\;\\ A_{-}e^{\Delta t/\tau_{-}},\;if\;\Delta t<0 \end{array}\right.\;$$

where $\Delta t=t_{j}-t_{i}$ is the time difference between post-synaptic and pre-synaptic spikes, and $\;A_{+},A_{-},\tau_{+}$ and $\tau_{-}$ are scaling and temporal constants. This mechanism reinforces synaptic connections for causal relationships Δt>0 and weakens them for reverse causation Δt<0. In the proposed model, STDP is incorporated into the base-policy network to dynamically refine its parameters during gameplay. By continuously adjusting synaptic weights, the network learns to prioritize actions that maximize cumulative rewards, a process formalized as:


$$\;{ℒ}_{STDP}=\sum_{(i,j)\in \varepsilon }\Delta w_{ij},$$

where *ε* represents the set of all synaptic connections. This dynamic weight adjustment augments the network’s capacity to respond to environmental changes, complementing the meta-policy’s broader adaptation. The combined use of MRL and STDP bridges the gap between global task-level adaptation and local decision-level refinement. MRL ensures that the agent rapidly adapts to new tasks by initializing effective parameters, while STDP fine-tunes these parameters in real-time to optimize performance within the task.

## 7. Conclusion

Through extensive experimentation and thorough testing, the MRL+STDP model has demonstrated its exceptional ability to swiftly adapt to new environments and effectively transfer learned behaviors from one context to another with minimal performance degradation. In comparison to traditional algorithms, it outperformed not only during the initial learning phase but, more crucially, in its capacity to adjust to previously unseen game environments without requiring further training. For example, while conventional models such as DQN and Double DQN experienced significant performance drops when applied to new games outside their training datasets, the MRL+STDP model exhibited stable performance, showcasing its robustness and flexibility.

A key strength of the MRL+STDP model lies in its ability to adjust synaptic weights dynamically using STDP, based on the timing of neuronal activations. This enhances its learning process and supports more refined strategy development. When combined with the rapid adaptation enabled by the meta-learning component, the model can effectively transfer knowledge from previous tasks to tackle novel challenges. This represents a major advancement over traditional models, which typically necessitate retraining or fine-tuning for new tasks.

The model’s superior performance goes beyond merely achieving higher game scores; it is also reflected in the sophistication of the strategies it employs. Analysis of gameplay evolution reveals that the MRL+STDP model develops intricate strategies, demonstrating a deeper understanding of game mechanics. This ability is crucial for practical applications where both adaptability and effective decision-making are essential in dynamic environments.

In addition to its performance, the MRL+STDP model’s efficiency in computational resource usage highlights its potential for use in environments with limited processing power. This, combined with its ability to learn quickly from minimal data, makes it well-suited for real-time decision-making scenarios such as autonomous driving, robotics, and financial systems. The broader implications of this study are significant, as the model’s principles can be applied to a variety of fields requiring rapid adaptation to new situations with minimal data. For instance, in healthcare, it could expedite the personalization of patient treatment plans, while in autonomous systems, it could improve robots’ interactions with their environments. The model’s capacity for hierarchical decision-making and managing continuous action spaces warrants further exploration, especially for applications in robotics and open-world simulations. Future research might also focus on optimizing the STDP mechanism to reduce computational demands without compromising learning efficiency, thus improving scalability for larger tasks. Additionally, integrating unsupervised pretraining could further enhance performance in environments where rewards are sparse or ambiguous.

## Appendix

Xavier initialization, also known as Glorot initialization, is a widely used method for weight initialization in deep neural networks. The method is designed to address issues related to the vanishing and exploding gradient problems that can arise when training deep networks. Specifically, it helps maintain the variance of the activations and gradients across layers, ensuring efficient backpropagation and improving the convergence rate during training. The Xavier initialization method works by setting the initial weights *W*_*l*_ of a layer *l* to be drawn from a uniform distribution with the following range:


$$\;W_{l}\sim 𝒰\left(-\sqrt{\frac{6}{n_{in}+n_{out}}},\sqrt{\frac{6}{n_{in}+n_{out}}}\right)$$

where *n*_in_ is the number of incoming neurons, and *n*_out_ is the number of outgoing neurons. Mathematically, for a neural network with *L* layers, the gradient update rule is:


$$\;{\Delta W}_{l}=-\eta \cdot \frac{\partial L}{\partial W_{l}}$$

where *η* is the learning rate, *L* is the loss function, and $\frac{\partial L}{\partial W_{l}}$ is the gradient with respect to the weights. If the variance of the weight distributions is not appropriately balanced (as it would be in random initialization), the gradients will either vanish or explode, leading to inefficient learning. By ensuring the proper variance using Xavier initialization, we allow gradients to flow more effectively, which speeds up convergence and stabilizes the learning process. In our experiments, we employed Xavier initialization due to its theoretical properties that help balance the weight distribution and mitigate the aforementioned issues.

To further illustrate the influence of weight initialization, we conducted experiments comparing the impact of different initialization methods (Xavier vs. random initialization) on the training process. We observed that Xavier initialization resulted in faster convergence in terms of episodes required to reach optimal performance, as shown by the faster drop in training loss and higher performance in the early stages of training. Additionally, we observed that networks with poorly initialized weights (e.g., small or large random values) exhibited erratic or slow training, sometimes requiring significantly more episodes to converge. On the other hand, Xavier-initialized networks demonstrated smoother training curves and were able to converge within a reasonable number of episodes, indicating both faster learning and more stable training dynamics.
